# Proton beam therapy in multimodal treatment for locally advanced squamous cell carcinoma of the nasal cavity and paranasal sinus

**DOI:** 10.1186/s13014-023-02296-3

**Published:** 2023-06-29

**Authors:** Takashi Saito, Masahiro Nakayama, Kayoko Ohnishi, Shuho Tanaka, Masatoshi Nakamura, Motohiro Murakami, Shin Matsumoto, Keiichiro Baba, Keitaro Fujii, Masashi Mizumoto, Keiji Tabuchi, Hideyuki Sakurai

**Affiliations:** 1 Department of Radiation Oncology, University of Tsukuba, Tsukuba, Japan; 2 Department of Otolaryngology, Head and Neck Surgery, University of Tsukuba, Tsukuba, Japan; 3 Department of Radiology, School of Medicine, International University of Health and Welfare, Narita, Japan

**Keywords:** Proton beam therapy, Squamous cell carcinoma, Nasal cavity, Paranasal sinus, Multimodal treatment, Outcomes

## Abstract

**Background:**

To evaluate proton beam therapy (PBT) in multimodal treatment for locally advanced squamous cell carcinoma of the nasal cavity and paranasal sinus (NPSCC).

**Methods:**

The cases in this study included T3 and T4 NPSCC without distant metastases that were treated at our center using PBT between July 2003 and December 2020. These cases were classified into 3 groups based on resectability and treatment strategy: surgery followed by postoperative PBT (group A); those indicated to be resectable, but the patient refused surgery and received radical PBT (group B); and those declared unresectable based on the extent of the tumor and treated with radical PBT (group C).

**Results:**

The study included 37 cases, with 10, 9 and 18 in groups A, B and C, respectively. The median follow-up period in surviving patients was 4.4 years (range 1.0-12.3 years). The 4-year overall survival (OS), progression-free survival (PFS), and local control (LC) rates were 58%, 43% and 58% for all patients; 90%, 70% and 80% in group A, 89%, 78% and 89% in group B; and 24%, 11% and 24% in group C. There were significant differences in OS (p = 0.0028) and PFS (p = 0.009) between groups A and C; and in OS (p = 0.0027), PFS (p = 0.0045) and LC (p = 0.0075) between groups B and C.

**Conclusions:**

PBT gave favorable outcomes in multimodal treatment for resectable locally advanced NPSCC, including surgery followed by postoperative PBT and radical PBT with concurrent chemotherapy. The prognosis for unresectable NPSCC was extremely poor, and reconsideration of treatment strategies, such as more active use of induction chemotherapy, may improve outcomes.

**Supplementary Information:**

The online version contains supplementary material available at 10.1186/s13014-023-02296-3.

## Background

Malignant tumors of the nasal cavity and paranasal sinus are rare tumors that account for about 3% of all head and neck (H&N) cancers [1]. There are various histologic types, with squamous cell carcinoma (SCC) being the most common and accounting for about half of such cases [2]. Because of their rarity and variable histology, many studies of treatment of malignant tumors of the nasal cavity and paranasal sinuses include various histological types. A meta-analysis showed the effectiveness of particle therapy for sinonasal cancer due to its relative physical and biological advantages over photon therapy [3]. However, only a few retrospective studies have focused on proton beam therapy (PBT) for sinonasal SCC [4, 5]. The largest prospective study of PBT included a wide variety of H&N and skull base malignancies, but the representation of cases specifically involving SCC was notably limited [6, 7].

There are various therapeutic options for sinonasal cancer, including surgery, radiotherapy, concurrent chemotherapy, and induction chemotherapy. The optimal combination of these treatments and the extent to which lesions are controllable using PBT are unclear. In this study, we evaluated outcomes and late adverse events of treatment of locally advanced SCC of the nasal cavity and paranasal sinus (NPSCC) using PBT in multimodal treatment.

## Subjects and methods

### Patients

Between July 2003 and December 2020, 41 patients with T3 and T4 NPSCC without distant metastases were treated at our center using PBT. This study included 37 of these patients, after exclusion of one patient who was not within the age range of 20–85 years and 3 patients with a follow-up period < 6 months, based on the study exclusion criteria. Staging was based on the 8th edition of the Union for International Cancer Control (UICC) TNM classification [8]. Because sphenoid sinus cancer stage is not defined in this edition, this was evaluated using the stage of nasal cavity and ethmoid sinus cancer. The patients were classified into 3 groups based on resectability and treatment modality: those who underwent surgery followed by postoperative PBT (group A); cases indicated to be resectable, but in which the patient did not wish to undergo surgery, and thus, received radical PBT without resection (group B); and cases declared unresectable and treated with radical PBT (group C). Resectability was determined at a multidisciplinary conference, and cases with T4b factors other than brain and dura mater involvement and severe pterygoid or pterygoid muscle invasion were judged to be unresectable.

### Proton beam therapy

PBT planning was performed using 2.5- to 5-mm slice computed tomography (CT). The patients were immobilized in the supine position with a thermoplastic mask. Passive scattering PBT plans were developed using VQA ver. 1.7 or 2.0 (Hitachi, Tokyo, Japan). The initial clinical target volume (CTV) was defined as CTV1, and CTV after treatment plan modification as CTV2. For radical PBT, CTV1 encompassed the gross tumor volume (GTV) plus a 5- to 10-mm margin and the adjacent nasal cavity and paranasal sinuses, and received a dose of 40–50 Gy (relative biological effectiveness, RBE). CTV2 was defined as GTV plus a 5-mm margin, to which an additional dose of 20–36 Gy (RBE) was administered. For postoperative PBT, CTV1 included the tumor bed and adjacent nasal cavity and paranasal sinuses, and received a dose of 50 Gy (RBE). In cases in which a micro-residual tumor was suspected after surgery, CTV2 was considered as the tumor bed and an additional dose of 10–16 Gy (RBE) was administered.

Dose constraints of 50 Gy (RBE) for the optic nerve, chiasma and brainstem, and 45 Gy (RBE) for the retina were used. However, when the tumor was adjacent to a critical organ, administering the dose to the CTV was prioritized. In cases where the brain received more than 60 Gy (RBE), the CTV dose was ensured while the irradiated volume was minimized as much as feasible. Patients without cervical lymph node metastases did not receive prophylactic neck irradiation.

### Chemotherapy

Concurrent chemotherapy was administered in cases in group A with suspected microscopic residual lesions and in all cases in groups B and C. Patients were treated with PBT alone if chemotherapy was contraindicated due to a poor performance status (PS) or severe comorbidities. Tegafur-gimeracil-oteracil (S-1) or a combination of 5-fluorouracil (5-FU) and cisplatin was used as the chemotherapy regimen before 2013, depending on the judgment of the attending physician, and cisplatin was used after 2014. S-1 was administered at 100 to 120 mg per day, depending on body surface area, for two weeks, followed by one week of rest. Two cycles of a combination of 5-FU and cisplatin were used as concurrent chemotherapy: 5-FU at 1000 mg/day continuously from day 1 to day 6, and cisplatin at 70 mg/m^2^ on day 1. Cisplatin alone was administered once every 3 weeks at a dose of 80 to 100 mg/m^2^, with three cycles given if possible. Induction chemotherapy was used only for cases in which tumor reduction was likely to improve resectability or contribute to preservation of function.

### Follow-up

Post-treatment evaluation was performed every 1–3 months during the first year and every 2–6 months thereafter. Follow-up examinations included CT, MRI, and positron emission tomography/CT as appropriate, in addition to physical examination and nasal endoscopy. If symptoms such as decreased vision appeared after PBT, an ophthalmologic exam was performed. Additionally, sites of initial recurrence were investigated and late adverse events were recorded using the Common Terminology Criteria for Adverse Events (CTCAE) ver. 5.0.

### Statistical analysis

Differences in patient background and treatment between groups were examined by Fisher exact test for nominal variables (sex, PS, smoking history, tumor location, T-stage, N-stage, chemotherapy regimen) and Kruskal-Wallis test for continuous variables (age, tumor volume, total PBT dose). Overall survival (OS), progression-free survival (PFS), and local-control (LC) rates were calculated from the start date of treatment. These rates were estimated using the Kaplan-Meier method and compared among groups A, B and C by log-rank test. The significance threshold for comparisons across all groups was set to 0.05, but the significance threshold for pairwise comparisons between groups was set to 0.0167 using the Bonferroni adjustment. All data were analyzed using JMP ver. 13 (SAS Institute, Cary, NC, USA).

### Ethical approval

This study was approved by the Ethical Review Committee and the University of Tsukuba Hospital Steering Committee (Tsukuba Clinical Research and Development Organization, R04-155). The study was conducted in accordance with the Declaration of Helsinki.

## Results

### Characteristics of patients and tumors

**Fig. 1:**
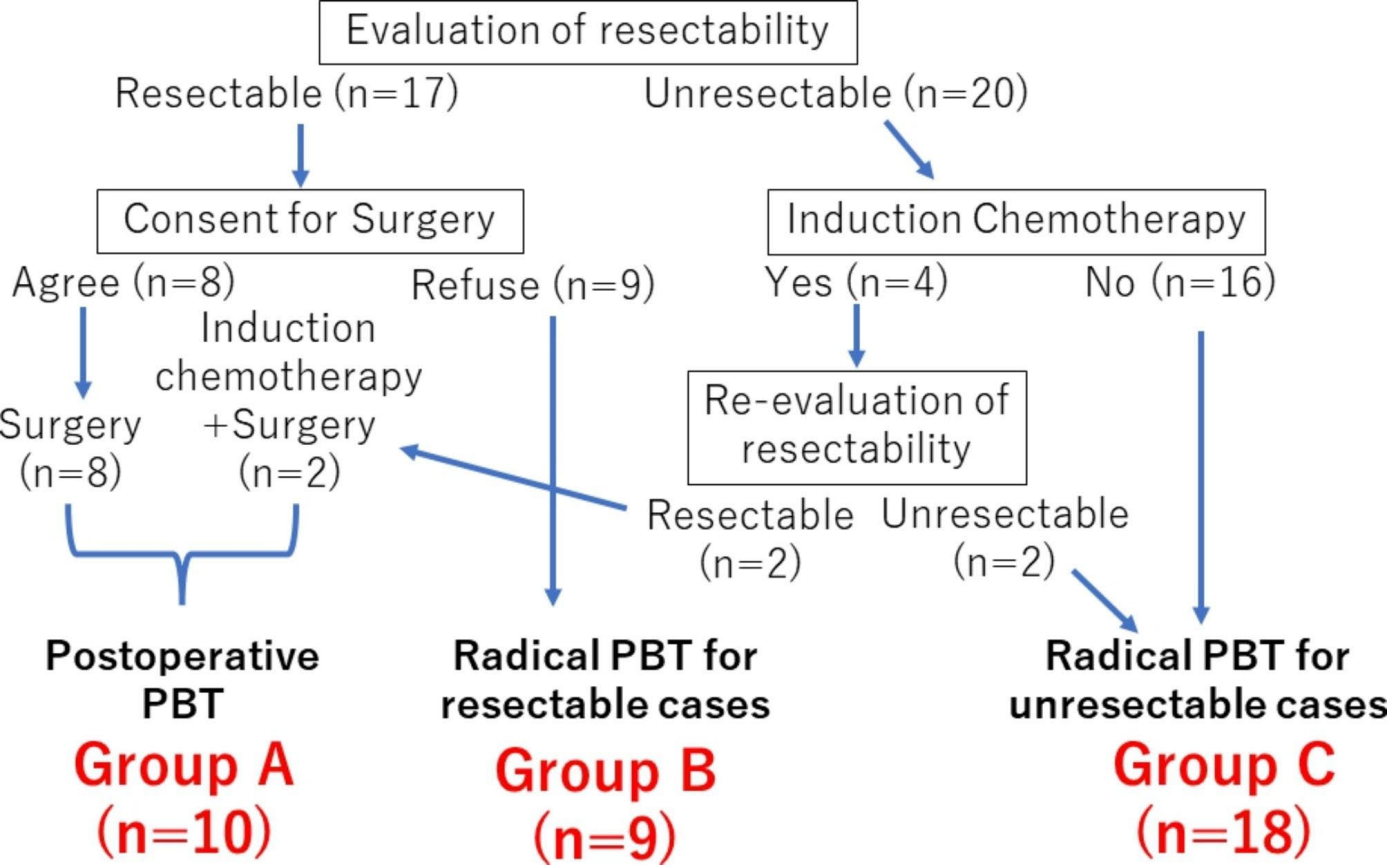
Treatment selection flowchart for groups A, B and C

The study included 37 patients, with 10 in group A, 9 in group B, and 18 in group C. The patient characteristics are listed in Table 1. Figure 1 shows a treatment selection flowchart for the three groups. The median age was 65 (range 32–81) years and 29 patients (78%) were male. The primary sites were the nasal cavity, maxillary sinus, ethmoid sinus and sphenoid sinus in 7 (19%), 15 (41%), 13 (35%) and 2 (5%) cases, respectively. The tumor stages of the primary site were T3, T4a and T4b in 4 (11%), 12 (32%) and 21 (57%) cases, respectively. Lymph node metastases were found in 2 patients (5%). Other than T-stage, the patient characteristics did not significantly differ among the groups.

**Table 1:** Patient characteristics

Characteristics		All (n = 37)	Group A (n = 10)	Group B (n = 9)	Group C (n = 18)	p-value
Age (year)	Median (range)	65 (32–81)	65 (32–81)	67 (57–77)	63 (44–81)	0.24
Sex	Male	29	8	8	13	0.78
	Female	8	2	1	5	
PS	0	20	8	4	8	0.3
	1	16	2	5	9	
	2	1	0	0	1	
Smoking history	Current	6	1	0	5	0.75
	Past	19	6	6	7	
	No Smoking	8	3	1	4	
	Unknown	4	0	2	2	
Location	Nasal	7	5	1	1	0.072
	Maxillary	15	2	6	7	
	Ethmoid	13	3	2	8	
	Sphenoid	2	0	0	2	
T-stage (UICC8th)	3	4	1	3	0	0.0006
	4a	12	6	4	2	
	4b	21	3	2	16	
N-stage (UICC8th)	0	35	10	8	17	0.5
	1	1	0	0	1	
	2b	1	0	1	0	
Tumor volume (cc)	Median (range)	46.1 (9.9-194.5)	42.2 (9.9-105.9)	40.8 (17.5-136.9)	46.3 (19.8-194.5)	0.49

PS: Performance status, UICC: Union for International Cancer Control

### Treatment details

Table 2 shows the treatment details. PBT was administered at 2 and 2.2 Gy (RBE) per fraction to 34 (92%) and 3 (8%) patients, respectively. A median total dose of 70 (range 50-81.4) Gy (RBE) was delivered in all cases, with medians of 66 (50–74) Gy (RBE) in group A, 70 (70–78) Gy (RBE) in group B, and 74 (70-81.4) Gy (RBE) in group C. In group A, 5 patients underwent extensive skull base tumor resection, dural resection and reconstruction; 3 underwent endoscopic nasal tumor resection; and 2 underwent total maxillary resection. In three of these cases, an orbital contentectomy was performed on the affected side. In all group A cases, at least a macroscopic gross total resection was achieved during surgery. Concurrent chemotherapy was administered in 22/37 cases (59%): 2/10 (20%) in group A, 8/9 (89%) in group B, and 12/18 (67%) in group C. Induction chemotherapy was used in 4 cases (11%), followed by surgery in 2 cases and radical PBT in 2 cases. The most common induction chemotherapy regimen was a combination of docetaxel, cisplatin and 5-FU, which was used in 3 cases.

**Table 2:** Details of treatment

Characteristics		All (n = 37)	Group A (n = 10)	Group B (n = 9)	Group C (n = 18)
Total dose (Gy, RBE)	Median (range)	70 (50-81.4)	66 (50–74)	70 (70–78)	74 (70-81.4)
Concurrent chemotherapy	Cisplatin	11	2	3	6
	S-1	7	0	4	3
	5-FU, cisplatin	4	0	1	3
	None	15	8	1	6
Induction chemotherapy	Docetaxel, cisplatin, 5-FU	3	2	0	1
	5-FU, cisplatin	1	0	0	1
	None	33	8	9	16
Surgery	Extensive skull base tumor resection, dural resection and reconstruction	5	5	0	0
	Endoscopic nasal tumor resection	3	3	0	0
	Total maxillary resection	2	2	0	0
	Biopsy only	27	0	9	18

RBE: relative biological effectiveness, 5-FU: 5-fluorouracil

### Survival and local control

**Fig. 2:**
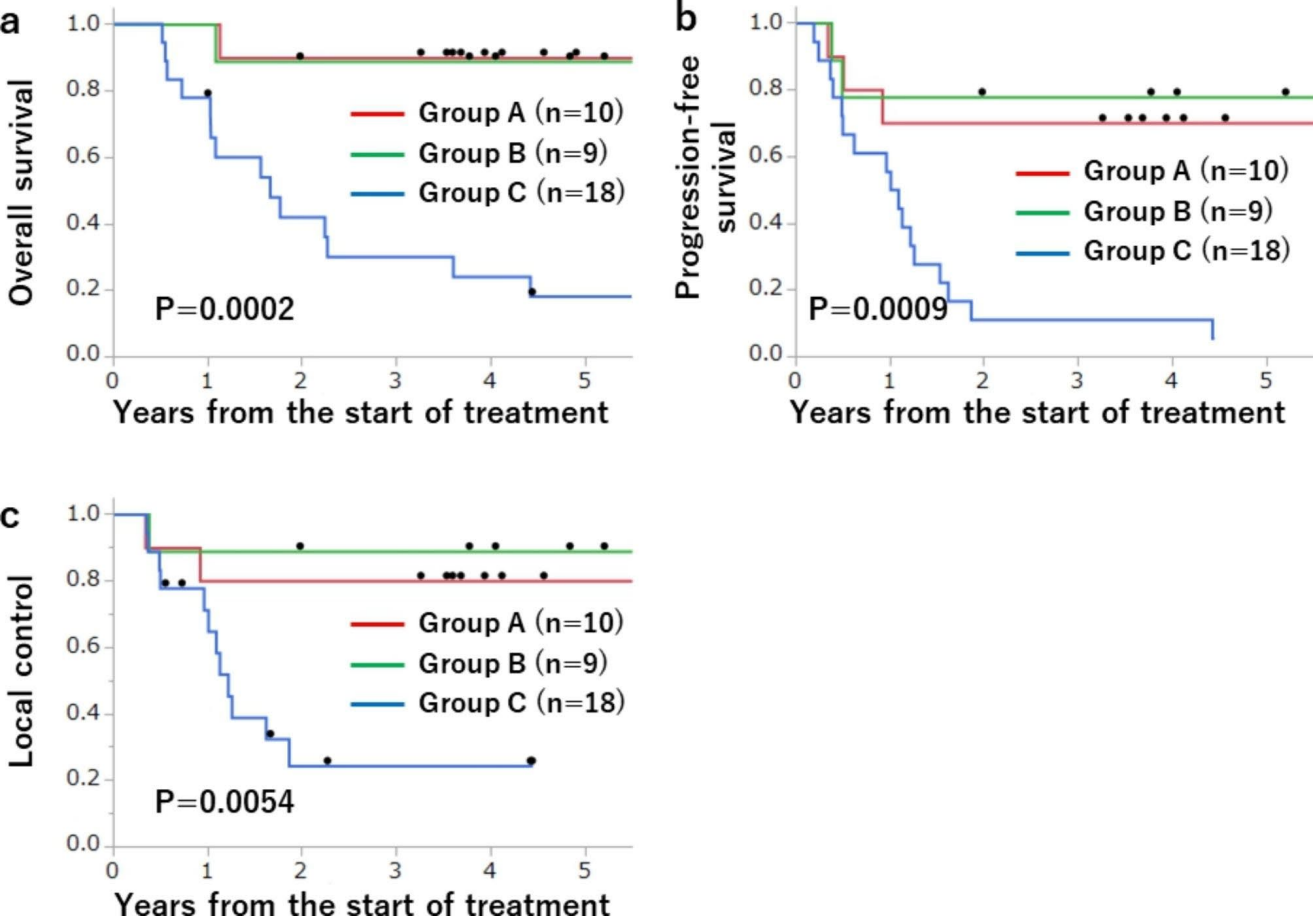
Kaplan-Meier curves for (a) overall survival, (b) progression-free survival, and (c) local control in groups A, B and C

At the last follow-up, 21 patients were alive, 15 had died of the primary disease, and 1 had died of another disease. The median follow-up period was 3.6 (range 0.5–12.3) years for all patients and 4.4 (1.0-12.3) years for the 21 surviving patients. Figure 2 shows Kaplan-Meier curves for groups A, B and C. The 4-year OS, PFS and LC rates were 58%, 43% and 58% for all patients; 90%, 70% and 80% for group A; 89%, 78% and 89% for group B; and 24%, 11% and 24% for group C. There were significant differences in outcomes between groups A and C (OS p = 0.0028, PFS p = 0.009) and between groups B and C (OS p = 0.0027, PFS p = 0.0045, LC p = 0.0075), but not between groups A and B (OS p = 0.91, PFS p = 0.75, LC p = 0.61).

### Recurrence patterns and salvage treatment

The patterns of recurrence were classified into six categories: local recurrence only (n = 11), local and regional recurrence (n = 2), local recurrence with distant metastasis (n = 2), regional recurrence only (n = 2), regional recurrence with distant metastasis (n = 1), and distant metastasis only (n = 3). Salvage treatments included maxillectomy (n = 2), neck dissection (n = 2), neck irradiation (n = 1), and chemotherapy (n = 6). Among these patients, one who underwent maxillectomy for local recurrence only and two who underwent neck dissection for regional recurrence only achieved recurrence-free survival. Details of cases with recurrence are summarized in Supplementary Table 1.

### Late toxicity

Grade 2 or severer late adverse events did not occur in group A, but occurred in 9 patients (33%) in groups B and C; however, there were no grade 5 adverse events. Details of the grade 2 or severer late toxicities are shown in Table 3. Decreased vision or retinopathy of grade 3 occurred in 4 cases and of grade 4 in 1 case, with times from the start of PBT to onset of symptoms of 2.2, 5.2, 3.3, 3.3 and 0.9 years, respectively. The other late adverse events included grade 2 (n = 1) and grade 3 (n = 1) central nervous system necrosis, grade 3 retinal vascular disorder (n = 1), and grade 2 zygomatic fracture (n = 1).

**Table 3:** Grade 2 or higher late adverse events

No	Age	Sex	Location	T-stage	Group	Dose (Gy, RBE)	Status	Survival (years)	Late adverse event	Grade	Onset from PBT (years)
1	68	M	Maxillary	3	B	70	Alive	4.8	Vision decreased (IpL)	3	2.2
2	67	M	Maxillary	4a	B	78	Alive	12.3	Vision decreased (IpL)	4	0.9
3	61	M	Ethmoid	4b	B	74	Alive	8.3	Retinopathy (IpL)	3	3.3
4	73	F	Maxillary	3	B	70	Alive	3.8	Retinopathy (IpL)	3	3.3
5	47	M	Nasal	4b	C	70	Alive	10.1	Retinopathy (IpL)	3	5.2
6	66	F	Maxillary	4a	C	74	Alive	7	Retinal vascular disorder (IpL)	3	4.2
7	64	F	Ethmoid	4b	C	76	Dead	1.7	Central nervous system necrosis	2	0.5
8	46	M	Ethmoid	4b	C	79.2	Dead	4.4	Central nervous system necrosis	3	1.5
9	53	F	Ethmoid	4b	C	79.2	Dead	3.6	Fracture	2	unknown

RBE: relative biological effectiveness, PBT: proton beam therapy, IpL: ipsilateral side

## Discussion

This study investigated outcomes of locally advanced NPSCC treated with PBT in multimodal treatment, stratified by resectability and treatment combinations. Cases in group A, which received the most curative treatment, had favorable results and no G2 or higher late adverse events. Those in group B, which were treated with high-dose radical PBT and concurrent chemotherapy for resectable cases, likewise had good outcomes and tolerable late adverse events, with comparable efficacy to that in group A. Conversely, in group C, the survival and local control rates of radical PBT for unresectable cases were low.

In comparison to photon therapy, a meta-analysis showed that particle therapy for sinonasal cancer reduces local recurrence. The rapid dose fall-off beyond the Bragg peak, which is a physical advantage of particle therapy, and the higher relative biological effectiveness of charged particle therapy, may contribute to improved outcomes [3]. A summary of previous studies on photon therapy and particle therapy for NPSCC is provided in Table 4. Despite a higher proportion of T4b cases in the PBT reports, ranging from 27 to 63% compared to 9–37% in photon therapy studies, LC rates were comparable, at 50–80% for PBT and 60–81% for photon therapy [4, 5, 9–14]. It can be challenging to make accurate comparisons due to the retrospective nature and small-scale of these studies. However, the fact that LC rates were maintained even with a higher percentage of T4b cases may suggest that the increased dose achievable with PBT might contribute to its potential greater efficacy than photon therapy for locally advanced NPSCC.

**Table 4:** Previous reports of photon therapy or particle therapy for squamous cell carcinoma of the nasal cavity and paranasal sinus

Author	Year	Number of patients	Cases with IC	Cases with surgery	Radiotherapy modality	Median dose	T4b cases	Median follow-up (years)	OS (year)	PFS (year)	LC (year)
Guan et al. [9]	2014	59	7%	39%	X-ray	66 Gy/33 fr 70 Gy/35 fr	24%	3.3	69% (3)	60% (3)	63% (3)
Duru Birgi et al. [10]	2015	43	7%	58%	X-ray	55 Gy/20 fr (R) 60 Gy/30 fr (PO)	14%	2.7	80% (2)	71% (2)	81% (2)
Park et al. [11]	2016	73	NR	29%	X-ray	72 Gy (R) 60 Gy (PO)	NR	NR	88% (2)	NR	69% (2)
									85% (5)		60% (5)
Pare et al. [12]	2017	68	59%	100%	X-ray	64 Gy/32 fr	9%	5.7	68% (2)	56% (2)	63% (2)
									58% (5)	53% (5)	63% (5)
Slevin et al. [13]	2021	56	21%	73%	X-ray	65 Gy/30 fr (R) 60 Gy/30 fr (PO)	NR	3.8	63% (3)	53% (3)	NR
Abdelmeguid et al. [14]	2021	123	100%	39%	X-ray	NR	37%	2.7	52% (3)	NR	NR
									44% (5)		
Russo et al. [4]	2016	54	2%	69%	Proton	72.8 GyE (including BID)	63%	6.8	67% (2)	57% (2)	80% (2)
									47% (5)	48% (5)	80% (5)
Toyomasu et al. [5]	2018	59	0%	0%	Proton Carbon	65 GyE/26 fr	27%	5.5	56% (3)	43% (3)	54% (3)
									42% (5)	35% (5)	50% (5)
Current study	2023	37	11%	27%	Proton	74 GyE/37 fr (R) 66 GyE/33 fr (PO)	57%	4.4	58% (4)	43% (4)	58% (4)

IC: induction chemotherapy, PO: postoperative, R: radical, NR: not reported, BID: bis in die, GyE: Gy equivalent

There are two previous reports on PBT for NPSCC, and one involved treatment combined with surgery or other therapies, with outcomes differing depending on resectability [4]. Although 63% of the cases in the previous report were T4b, surgery was performed in 69%, with about half of these cases resulting in incomplete resection. Cases that underwent gross total resection were found to have a significantly higher 5-year OS rate than those that underwent partial resection or biopsy alone (77% vs. 31%). This finding is similar to our results; however, the current study presents a new finding since we analyzed a separate group of patients who received radical PBT with concurrent chemotherapy in resectable cases. In contrast, Toyomasu et al. found that resectability did not have a significant effect on outcomes in particle therapy alone for NPSCC [5]. The cause of the different results is unclear, but may be attributable to differences in treatment strategies. In our study, resectability had a substantial impact on outcomes because a large percentage of cases were treated with PBT combined with multimodal treatment, such as surgery and chemotherapy. Thus, this study shows that radical PBT with concurrent chemotherapy is a promising option for resectable cases, especially when surgery is not acceptable. However, the higher incidence of late adverse events compared to surgery followed by PBT should be taken into consideration, and if resection is acceptable, surgery may still be the preferred option. Future use of intensity-modulated proton therapy may further reduce the risk of late toxicities associated with radical PBT.

In this study, outcomes for unresectable NPSCC were unfavorable, but there is a possibility of improving the prognosis if induction chemotherapy can render these cases resectable. T4b cases are generally considered unresectable, but a database study of T4b NPSCC showed that cases treated with surgery plus postoperative irradiation had a significantly better prognosis than those treated with radical irradiation (5-year OS 42.5% vs. 21.7%) [15]. To improve resectability in T4b cases, induction chemotherapy is a promising option for locally advanced NPSCC [14, 16, 17]. The response rate to platinum-based induction chemotherapy is reported to be 62–83%, and this treatment is likely to improve resectability. Several studies have reported OS of 68–77% for induction chemotherapy responders, compared to 25–36% for non-responders [14, 18, 19]. Among the 20 unresectable cases in our study, induction chemotherapy was administered to only 4 patients. Interestingly, two of these patients underwent surgery and postoperative PBT, which resulted in favorable outcomes. In the future, it will be important to consider use of induction chemotherapy to improve the prognosis in initially unresectable cases.

In regard to late adverse events following PBT, the principal complications observed in this study were deceased vision including retinopathy and central nervous system necrosis, aligning with previously reported incidence [20]. These adverse events stem from the anatomical proximity of lesions to the retina, optic nerve, and brain, which, in some cases, can be difficult to avoid even with PBT. Radiation-induced skin toxicity (RIST) is a concern with PBT, given the absence of build-up region unlike photon therapy. However, severe late RIST was not observed in this study, which is consistent with previous finding that PBT does not conclusively increase RIST [21]. It should be noted, that this study is retrospective, and minor late adverse events may not have been fully assessed. Additionally, the reported ability of PBT to minimize impacts on dental and facial bones represents a particular benefit [22]. This study only identified a single case of minor fractures, further demonstrating this benefit. While it is known that dysphagia can occur as a late adverse event after radiotherapy for H&N cancer, the omission of prophylactic neck irradiation in this study also helped mitigate this occurrence [23].

A limitation of this study is its retrospective design and small sample size, which prevented exclusion of possible confounding factors. However, the rarity of NPSCC limits the number of cases treated at a single center. An increased number of cases could have been included by permitting different histologic types, but the study was limited to SCC because the characteristics of this disease vary greatly according to histologic type. Thus, homogeneity of the cases was maintained, but this reduced the statistical validity. It’s a matter of ongoing debate as to which types of disease can be more effectively treated with PBT as opposed to photon therapy [24]. In Japan, the efficacy of PBT for NPSCC has been recognized and was approved for national insurance coverage in 2018, thus an increase in the number of cases is anticipated. However, as the number of PBT facilities is currently limited, establishing a comprehensive data collection system for clinical data is of critical importance [25]. The number of PBT facilities in Japan is increasing and a more accurate analysis should be possible using cases in a multicenter study in the future.

## Conclusion

PBT in multimodal treatment, including surgery followed by postoperative and radical PBT with concurrent chemotherapy, showed favorable outcomes for resectable locally advanced NPSCC. The prognosis for unresectable NPSCC was extremely poor, and reconsideration of treatment strategies, such as more active use of induction chemotherapy, may improve outcomes.

## Electronic supplementary material

Below is the link to the electronic supplementary material.

Supplementary Material 1

## Data Availability

The datasets used and/or analyzed during the current study are available from the corresponding author on reasonable request.
